# Teaching critical thinking about health using digital technology in lower secondary schools in Rwanda: A qualitative context analysis

**DOI:** 10.1371/journal.pone.0248773

**Published:** 2021-03-22

**Authors:** Michael Mugisha, Anne Marie Uwitonze, Faith Chesire, Ronald Senyonga, Matt Oxman, Allen Nsangi, Daniel Semakula, Margaret Kaseje, Simon Lewin, Nelson Sewankambo, Laetitia Nyirazinyoye, Andrew D. Oxman, Sarah Rosenbaum

**Affiliations:** 1 Institute of Health and Society, Faculty of Medicine, University of Oslo, Oslo, Norway; 2 School of Public Health, College of Medicine and Health Sciences, University of Rwanda, Kigali, Rwanda; 3 Tropical Institute of Community Health and Development, Kisumu, Kenya; 4 Department of Medicine, Makerere University, College of Health Sciences, Kampala, Uganda; 5 Centre for Informed Health Choices, Norwegian Institute of Public Health, Oslo, Norway; 6 Faculty of Health Sciences, Oslo Metropolitan University, Oslo, Norway; 7 Health Systems Research Unit, South African Medical Research Council, Cape Town, South Africa; National Taiwan University of Science and Technology, TAIWAN

## Abstract

**Introduction:**

Adolescents encounter misleading claims about health interventions that can affect their health. Young people need to develop critical thinking skills to enable them to verify health claims and make informed choices. Schools could teach these important life skills, but educators need access to suitable learning resources that are aligned with their curriculum. The overall objective of this context analysis was to explore conditions for teaching critical thinking about health interventions using digital technology to lower secondary school students in Rwanda.

**Methods:**

We undertook a qualitative descriptive study using four methods: document review, key informant interviews, focus group discussions, and observations. We reviewed 29 documents related to the national curriculum and ICT conditions in secondary schools. We conducted 8 interviews and 5 focus group discussions with students, teachers, and policy makers. We observed ICT conditions and use in five schools. We analysed the data using a framework analysis approach.

**Results:**

Two major themes found. The first was demand for teaching critical thinking about health. The current curriculum explicitly aims to develop critical thinking competences in students. Critical thinking and health topics are taught across subjects. But understanding and teaching of critical thinking varies among teachers, and critical thinking about health is not being taught. The second theme was the current and expected ICT conditions. Most public schools have computers, projectors, and internet connectivity. However, use of ICT in teaching is limited, due in part to low computer to student ratios.

**Conclusions:**

There is a need for learning resources to develop critical thinking skills generally and critical thinking about health specifically. Such skills could be taught within the existing curriculum using available ICT technologies. Digital resources for teaching critical thinking about health should be designed so that they can be used flexibly across subjects and easily by teachers and students.

## Background

We are confronted all the time with claims about the world. Many of these claims are not directly testable by most of us. We must figure out how to evaluate other people’s arguments to come to our own conclusions, particularly about causal claims [1]. Adolescents, like adults, encounter a wide range of health-related claims in their daily lives, and many of those are claims about health interventions, i.e., statements or messages about purported benefits or harms of actions people can take to protect or improve health. When confronted with such claims, most people are not trying to be scientists. Rather, they are trying to figure out what to believe and what to do.

Such claims are obtained from peers, families, the community, social and mass media. Misleading claims can lead to bad decisions about health, if they are believed. For example, there are endless claims about what people can do to prevent or treat COVID-19 [2]. Acting on unreliable claims can lead to unnecessary suffering and wasted resources [3–7]. Conversely, failure to believe and act on reliable claims about health interventions also leads to unnecessary suffering and inefficient use of health services [8–10].

Making good decisions about health depends on critical thinking, people’s ability to obtain, process and understand health information needed to make informed decisions [11–14]. Additionally, people need to think critically about health information, for instance to assess the trustworthiness of claims about health interventions or to understand how to deal with conflicting claims [15]. Many countries have moved towards competence-based curricula and include critical thinking as a key competence [16, 17], although not specifically critical thinking about health. A strong case can be made for investing in health education for adolescents based on developmental science [18]. However, few educational interventions to improve adolescents’ ability to think critically about health have been evaluated rigorously [19].

We are a team developing and evaluating resources to enable young people to think critically about health claims. The team includes researchers from East Africa, where the resources are being developed and evaluated, as well as from Chile and Norway. The team is part of the Informed Health Choices (IHC) network, which includes researchers from over 20 countries who are developing and testing learning resources for primary and secondary schools [20].

We first identified key concepts (principles) that people need to understand and apply when deciding what health claims to believe and what to do [21]. Together with teachers in Uganda, we prioritised concepts that were relevant for primary school children [22]. We have also prioritised concepts for secondary schools, together with national curriculum committee members and teachers in Rwanda, Uganda, and Kenya [23]. We developed and tested learning resources in Ugandan primary school children [24, 25]. In a follow up study, we showed that children retained what they had learned for at least one year [26]. The team has translated primary school learning resources to Kinyarwanda and Kiswahili and piloted their use in Rwanda and Kenya. Key findings from the Rwandan pilot study indicated that IHC resources were useful and feasible to use in Rwandan primary schools [27]. The primary school resources have also been translated to other languages, including Chinese, Croatian, French, Greek, Italian, Norwegian, Persian, Portuguese, Spanish and pilot testing of translated resources is ongoing in several countries [28].

In a process evaluation, researchers found that lack of time in the curriculum and printing costs were major challenges to scaling up use of the IHC primary school resources [29]. One way of reducing the cost of the intervention would be to use digital resources. Digital learning resources are much cheaper to distribute than printed resources because they eliminate printing costs, and they do not need to be physically shipped. However, schools may not be equipped to use digital resources and teachers and students may prefer printed learning materials. Further, we conducted a context analysis in Norway to explore the demand for teaching critical thinking about health in primary schools [30]. We found that although teachers were interested, there was little time available for teaching new content outside the curriculum and little time for teachers to seek out and test new resources.

Building on what we learned in our work with primary school resources, and in collaboration with stakeholders in education, we are developing digital learning resources for secondary school students in East Africa that can be easily adapted for use in other countries. To inform the development of the resources and ensure that they are well suited for the Rwandan context, we conducted a context analysis to explore 1) the demand for learning resources, 2) the extent to which these fit with the curriculum and 3) ICT conditions in secondary schools. Researchers in Kenya and Uganda carried out similar context analyses [31–33]. While our focus is on understanding the context for developing suitable learning resources for critical thinking about health, our findings can also inform the design of other digital learning resources in low resource educational settings.

## Methods

We used a qualitative descriptive study approach [34]. This entails describing a phenomenon without moving far from or into the data; it requires less interpretation than an “interpretive descriptive” approach. We chose this method because the nature of the data we sought was primarily factual. We employed four qualitative methods: document review, key informant interviews, focus group discussions, and observations.

### Document review

The document review included analysis of the existing curriculum, of approved learning resources in lower secondary schools, and of current documentation on ICT for education (ICT for education policy, ICT implementation plans, and guidelines for use of ICT in education). We searched for relevant documents on the official websites of the Rwanda Education Board (REB) and Ministry of Education. We consulted REB to retrieve and obtain clarifications of documents that could not be found on the official website. In total, we reviewed 29 documents for curriculum, resources and ICT use in Rwanda.

We reviewed the national curriculum for lower secondary schools. We read syllabuses for each subject taught in lower secondary schools. For each subject, we reviewed its rationale, competences, objectives, topic areas and units taught. We explored what health topics are covered in the curriculum and in which subjects and course units these health topics are located. We reviewed how critical thinking is generally covered in the curriculum and specifically in relation to health topics. We mapped if there were any IHC concepts and competences reflected in the curriculum. We used the IHC Key Concepts as a framework for reviewing the curriculum, mapping where in the curriculum IHC concepts are relevant explicitly or implicitly. The IHC Key Concepts includes 49 principles grouped in three categories, each with three high level concepts, and corresponding competences (see Table 1). We did not review international or special needs curricula used in Rwandan lower secondary schools.

**Table 1:** IHC key concepts that formed a framework for curriculum document analysis.

No	Short description of IHC concepts for critical thinking about treatments	Informed Health Choices Competence
**1**	**Claims concepts**	
1.1	It should not be assumed that treatments are safe or effective—or that they are not.	Recognise when a claim has an untrustworthy basis
1.2	Seemingly logical assumptions are not a sufficient basis for claims.	
1.3	Trust in a source alone is not a sufficient basis for believing a claim.	
**2**	**Comparison concepts**	
2.1	Comparisons of treatments should be fair.	Recognise when evidence used to support a treatment claim is trustworthy or untrustworthy
2.2	Syntheses of studies need to be reliable.	
2.3	Descriptions should clearly reflect the size of effects and the risk of being misled by the play of chance.	
**3**	**Choices concepts**	
3.1	Problems and options should be clear.	Make well-informed decisions about treatments
3.2	Evidence should be relevant.	
3.3	Expected advantages should outweigh expected disadvantages.	

We reviewed e-books approved by REB. We started by reviewing all books used in lower secondary schools of Rwanda. For each electronic book used in lower secondary schools, we reviewed whether the content included health topics or critical thinking about health.

We reviewed existing documentation on ICT use in secondary education, including existing national policy for use of ICT in education, and strategic and implementation plans for ICT in secondary schools. We also reviewed existing e-learning platforms and digital learning resources available through the REB gateway. We explored the status of the rolling out of ICT infrastructure in Rwandan secondary schools, and the availability of resources (equipment, Internet access, e-learning content, etc) in schools where ICT has been rolled out.

### Key informant interviews

We interviewed key informants such as curriculum development and ICT for education at REB, secondary school teachers, and school ICT support officers. We explored how the competence-based curriculum is implemented in Rwanda, focusing on critical thinking and health topics, and how competence-based learning is evaluated. We asked secondary school teachers and ICT support officers at schools to describe how they teach competence-based curriculum with a focus on critical thinking and health related topics. We also explored ICT use for teaching and learning, and challenges using digital learning resources.

### Focus group discussions

We conducted focus group discussions with students to explore how they obtain health information, what they use as a basis for making health decisions, and claims they hear in everyday life. We explored whether critical thinking about health is something they would be interested to learn in school. We also explored how they search for information about health and other topics at school. Finally, we explored how they access and use ICT for learning in school.

### Observation

We visited selected schools and observed what ICT infrastructure is available and how it is used for teaching and learning. We observed existing ICT labs, digital equipment, Internet access, and content. Where we were able to access ongoing classes, we observed how ICT was used in teaching and learning.

### Sampling

First, we sampled documents to review according to the objectives. We purposively selected curriculum documents, approved learning resources and ICT policy and implementation documents (n = 29). For the curriculum and learning resources we selected those used in lower secondary schools in Rwanda. Second, we used convenience sampling to select five schools to conduct observations, interviews with teachers, and focus group discussions with students. Due to time and budget constraints, we applied convenience sampling to select five schools. We took care to choose schools that varied as much as possible in terms of ownership (private/public), day/boarding, equipment, and location (urban/rural). In each school, the school administration identified at least 10 students from lower secondary school with whom we conducted a focus group discussion. Two of the five focus group discussions were conducted out of school premises due to the Covid-19 pandemic. In each school, we purposively selected two to three teachers of biology and English because the current curriculum informed us that health topics were mainly taught in those subjects. We also interviewed people in charge of ICT at each school. Lastly, we purposively selected 5–10 key informants from REB’s departments of curriculum development and ICT for education. In order to capture the opinions, views and experiences of a wide range of participants, we selected participants that were of direct relevance to our study objectives.

### Data collection procedures

For the document review, we used the study objectives and IHC Key Concepts as frameworks for collecting data. We extracted statements pertinent to each study objective. We summarised all findings in a single table, including the name of the document, the extracted statement, and the page number where the statement was found. This exercise was done independently by two researchers who then compared the data they extracted and resolved any disagreement through discussion.

For key informant interviews, we used semi-structured interview guides to collect information from the study participants, one for teachers and one for policy makers. Guides included questions that covered critical thinking about health, resources for teaching critical thinking, and ICT infrastructure used in teaching and learning. Guides also explored existing challenges and opportunities for using ICT for teaching and learning. We piloted the two interview guides with a few participants first and slightly modified them as needed. We interviewed participants face to face in a private place of their choice. Participants were encouraged to express their views freely and take discussion in a new relevant direction. We conducted some interviews with two or three teachers or REB key informants at the same time.

We also used an interview guide to conduct focus group discussions with students. We asked questions to explore how they learn to think critically, what claims about treatment effects they are familiar with, which sources of health information they use, and how they use ICT for learning purposes. We approached and conducted interviews at the workplace of study participants in a designated room that assured privacy of participants and recording of discussions. Interviews and focus group discussions were moderated by a male PhD fellow with Master of Public Health and experience qualitative research (first author). Each interview lasted at least an hour and the focus group discussion lasted between one hour and half. At least two researchers conducted each interview and focus group discussion. One person guided the discussion, and another took notes and recorded the discussion. Interviews and focus group discussions were recorded, transcribed verbatim and translated to English if the interview was conducted in Kinyarwanda. We collected observations using a checklist that covered ICT equipment, internet-connectivity, and e-learning content used in schools.

The amount of data we collected was guided by considerations of the variation in issues emerging from the data and the extent to which we were able to explain these variations. We considered our time and resource constraints and the need to avoid large volumes of data that cannot be easily managed or analysed as highlighted in the literature [35, 36].

### Data analysis

We compiled and analysed all data from the document review, key informant interviews, focus group discussions, and observations together, using a framework analysis approach for applied research [37]. This approach differs from thematic content analysis in that it is deductive in nature with pre-set objectives [38]. It also involves analysing, classifying and summarising data in a thematic framework [39]. We began by reading all notes, transcripts, and documents to familiarise ourselves with the data. Then we conducted an analysis based on a coding scheme of initial themes derived directly from the objectives of our study: 1) demand for learning resources to teach critical thinking about health, 2) links between critical thinking about health and the curriculum, and 3) current and expected ICT conditions for teaching and learning in secondary schools. We determined sub-themes from data within each initial theme. We indexed all the data using the initial themes and sub-themes and rearranged data within and across themes (charting) to compare summaries of data during analysis. Two researchers independently analysed the data and compared their findings. The two researchers discussed disagreements in codes and themes and agreed on the final themes.

We summarized the key findings and assessed our confidence in these using a version of the Confidence in the Evidence from Reviews of Qualitative research (GRADE-CERQual) approach [40]. GRADE-CERQual was modified for primary qualitative studies [29, 41]. GRADE-CERQual is a systematic and transparent method for assessing the confidence in evidence from reviews of qualitative research through the lens of four components: methodological limitations, data adequacy, coherence and relevance [42]. Although CERQual has been designed for assessing findings emerging from qualitative evidence syntheses, the components of the approach are also suitable for assessing findings from a single study with multiple sources of qualitative data. We modified the components slightly as follows: 1) Methodological limitations: the extent to which there are concerns about the sampling and collection of the data that contributed evidence to an individual finding, 2) Coherence of the finding: an assessment of how clear and compelling the fit is between the data and the finding that brings together these data, 3) Adequacy of the data contributing to a finding: an overall determination of the degree of richness and quantity of data supporting a finding and 4) Relevance: the extent to which the body of evidence supporting a finding is applicable to the context (perspective or population, phenomenon of interest, setting) specified in the study question.

Two authors applied the modified GRADE-CERQual approach to each study finding and made a judgement about our overall confidence in the evidence supporting the finding. We judged confidence as being high, moderate, low, or very low. All findings started as high confidence and were graded down if there were important concerns regarding any of the components described above [43].

### Ethical considerations

The study was performed in accordance with the protocol and regulatory requirements, guidelines, and principles for conducting studies involving human subjects in Rwanda. Ethical clearance was obtained from the Rwandan National Ethics Committee (RNEC) for the entire informed health choices project (approval number 916/RNEC/2019). Study participants signed a written informed consent before participating in the study. Students under the age of 18 signed assent forms and consent was obtained from their corresponding school administration at school.

## Results

We reviewed 29 documents related to the curriculum, syllabuses, textbooks, and ICT for education in Rwanda. We interviewed 27 key informants, including policymakers, and teachers. We conducted five focus group discussions with groups of nine to 11 students, and we made observations in five schools. Characteristics of the schools, students, teachers, and policymakers are summarised in Table 2. We categorised our findings in themes and sub-themes as described below. CERQual assessments are in parentheses.

**Table 2:** Demographic characteristics of schools visited, and participants interviewed.

**Schools characteristics**	**Number (n = 5)**
**Ownership**	
Public	2
Private	1
Public/private	2
**School type**	
Day school	2
Boarding school	3
**Students characteristics**	**Number (n = 51)**
**Age**	
13–15 years	43
16–18 years	8
**Gender**	
Male	18
Female	33
**Teachers characteristics**	**Number (n = 19)**
**Subject taught**	
Sciences	13
Languages	6
**Gender**	
Male	15
Female	4
**Policymakers characteristics**	**Number (n = 8)**
**Gender**	
Male	5
Female	3
**Work domain**	
Curriculum	4
ICT for education	3
Stakeholder in education	1

### Demand for resources to teach critical thinking about health

#### Demand in the curriculum

The competence-based curriculum requires that students develop generic competences including critical thinking, research and problem solving in all subjects (high confidence). In 2016, Rwanda switched from a knowledge-based curriculum to a competence-based curriculum. The current curriculum emphasises developing learners’ knowledge, skills, and attitudes that together build competences needed in real life. It also places the learner at the centre of teaching and learning processes. The learner is considered a source of information and is expected to drive learning processes, while the teacher’s role is to guide.

*“The former curriculum was objective-based*, *where the teacher was the source of everything*, *He/she was the one teaching students*, *providing all the information*, *and students could write all that the teacher said*, *But now in the current competence-based curriculum*, *the focus is more on learners*, *where students participate more in learning and teaching process than the teacher himself*.*”*

**Policymaker 03**

The current curriculum aims for learners to develop generic competences that promote higher order thinking skills. These competences are expected to impart learners with understanding of subjects and skills needed in the job market, as well as to promote life-long learning. The curriculum describes generic competences that include critical thinking, research, and problem solving.

In developing critical thinking competence, learners are expected to demonstrate that they “*think reflectively*, *broadly and logically about challenges encountered in all situations*, *weigh up evidence and make appropriate decisions based on experience and relevant learning*, *think imaginatively and evaluate ideas in a meaningful way before arriving at a conclusion and explore and evaluate alternative explanations to those presented by others*.*”* Similarly, for research and problem-solving skills competence, learners should *“be resourceful in finding answers to questions and solutions to problems*, *produce new knowledge based on research of existing information and concepts and sound judgment in developing viable solutions*, *explain phenomena based on findings from information gathered or provided*.*”* ***Rwanda Curriculum framework***, page 11.

According to the curriculum, these generic competences and others must be reflected and developed in all subjects taught in lower secondary schools in Rwanda.

The current curriculum lays out the demand for development of new textbooks and teachers’ guides to facilitate a learner-centred approach (high confidence). REB’s department of curriculum and material production is developing learning resources for each subject to increase the availability of such resources in schools.

*“The learner-centred approach required for the new curriculum demands a variety of teaching and learning textbooks and resources*, *Teachers’ guides for textbooks and the National Curriculum Syllabuses will provide subject teachers with advice and guidance on effective strategies for teaching their subjects and for optimising students’ progress in terms of subject knowledge*, *skills*, *attitudes and competences*.*”*

***Rwanda curriculum framework*, *page 24***.

#### Demand for critical thinking learning resources in subjects taught in lower secondary schools

Health related topics taught in secondary school subjects provide opportunity for developing competences for critical thinking about health among learners (high confidence). We explored all subjects in the lower secondary curriculum to determine where health topics are covered. Among 14 subjects taught in lower secondary schools, three subjects (biology and health sciences, home science, and English) covered health topics in their syllabuses. Broad health themes are included, such as sexual and reproductive health, infectious and non-infectious diseases, food and nutrition. Table 3 provides an overview of which subjects and units in the curriculum cover health topics.

**Table 3:** Units covered in lower secondary school that teach health.

Subject	Units
**Biology and health sciences**	• Classification of diseases. • Human reproductive system.
	• Reproduction, pregnancy and childbirth • Puberty and sexual maturation.
	• Sexual behaviour and sexual responses • Immunity and vaccination
	• Infectious and non-infectious diseases. • Pregnancy prevention
	• Reducing risks of STI and HIV • Social factors that affect good health
	• Decision making regarding sexual relationship
	• HIV and AIDS, stigma, treatment, care and support.
**Home Science**	• Personal health and etiquettes.
**English**	***Oral and written communication***
	•Food and nutrition • Health
	• Diet and health • Traditional beliefs and practices

In reviewing the content and activities for health-related topics, we found opportunities for teaching critical thinking about health. In addition, statistics and probability, which are taught in mathematics are linked to concepts for critical thinking about health research.

We did find some competences of biology, chemistry, mathematics subjects that aligned with competences in the IHC Key Concepts framework. These competences are rooted in generic competences described in the curriculum framework. They include “critical thinking, research and problem solving, creativity and innovation, communication, lifelong learning, cooperation, interpersonal relations, and life skills.” Specific broad competences in the syllabuses for subjects are based on these generic competences (see Table 4). The learner studying those subjects is expected to appreciate that science is evidence-based and should apply science in real life to make good choices and find solutions. Students use small-group discussions to conduct class activities and reflect on content delivered in class, a learning strategy that is aligned with critical thinking. At the end of lower secondary school, students should be able to apply science in advocating for personal, family and community health (high confidence).

**Table 4:** Links between the Rwandan lower secondary school curriculum and concepts and competences in the informed health choices key concepts framework.

IHC competances	Corresponding IHC concept categories and sub-categories	Competences in the Biology (B), Chemistry (C) and Mathematics (M) curricula
**Recognise when a claim has an untrustworthy basis**	**Claims**	**Recognise that science is evidence based** and understand the usefulness and limitations of a scientific method (B).
	• It should not be assumed that treatments are safe or effective—or that they are not.	
	• Seemingly logical assumptions are not a sufficient basis for claims.	**Develop attitudes on which scientific** investigations depend, such as honesty, persistence, critical thinking and tolerance of uncertainty (C, M).
	• Trust in a source alone is not a sufficient basis for believing a claim.	
		**Analyse scientific phenomena** relating to real life experiences (B, C, M).
		**Acquire sufficient knowledge** and understanding to use ICT skills effectively to enhance learning and communication to become confident citizens in a technological world and develop an informed interest in scientific matters (B)
		**Apply the knowledge of chemistry** to make scientifically informed decisions on the choice of chemical products on the market (C).
**Recognise when evidence used to support a treatment claim is trustworthy or untrustworthy**	**Comparisons**	**Use the principles of scientific methods** and the application of experimental techniques to solve specific problems (B, C).
	• Comparisons of treatments should be fair.	
		**Apply acquired knowledge in Mathematics** to solve problems encountered in everyday life (M).
	• Syntheses of studies need to be reliable.	
		**Interpret simple diagrams and statistics**, recognizing the ways in which representations can be misleading (M).
	• Descriptions should clearly reflect the size of effects and the risk of being misled by the play of chance.	
**Make well-informed decisions about treatments**	**Choices**	**Recognise that science is evidence based** and understand the usefulness and limitations of a scientific method (B).
	• Problems and options should be clear.	
		**Develop attitudes on which scientific** investigations depend, such as honesty, persistence, critical thinking and tolerance of uncertainty (C, M).
	• Evidence should be relevant.	
	• Expected advantages should outweigh expected disadvantages.	
		**Analyse scientific phenomena** relating to real life experiences (B, C, M).

Students should be able to *“*…*apply basic mathematical concepts*, *principles and processes to solve problems; analyse and explain scientific phenomena relating to real life experience; use and experiment with a range of scientific and technological tools and equipment and draw appropriate conclusions; advocate for personal*, *family and community health*, *hygiene and nutrition*…*”*

***Rwanda curriculum framework*, *page 14***.

#### Teachers’ needs in relation to resources to teach critical thinking about health

Understanding and developing critical thinking about health varies among teachers (moderate confidence). The teachers we interviewed noted that they understand critical thinking as a way of reflecting on class lectures through discussion among learners. Some teachers we interviewed also develop research and problem-solving skills by encouraging learners to search the Internet and books to get further information beyond what is taught in class. Other teachers understand critical thinking as a way of reflecting on topics learned in class and how these apply in real life.

*“We give them health topics to search on the Internet or in books*, *They discuss in class and present* [what they find] *during debates*.**”**

**English teacher**

*“For example*, *we teach infectious and non-infectious diseases*, *We can ask them some diseases they see at home*, *we ask a nurse to explain these diseases*, *so they think beyond class and get understanding of what infectious diseases are*.”

**Biology and health sciences teacher**

We interviewed five staff from the REB curriculum department to explore the need to develop learning resources to teach critical thinking about health. They noted that, in their view, teachers have little experience in teaching critical thinking and other new competences. This, they stated, is because most teachers have been trained in the previous knowledge-based curriculum. They also noted that teachers have different understandings of what is meant by critical thinking, and their competences vary. The curriculum department staff suggested that teachers do not know how to develop their competences in this area, and that there are no learning resources to help them.

*“Critical thinking is reflected in the curriculum but teaching it is still problematic because understanding of teachers for critical thinking varies and some don’t even understand it*, *Yes*, *you need to develop critical thinking*, *but how do you do it and what materials do you use*? *Which books do you use*? *You see it is a problem*.*”*

**Policymaker**

#### Students’ needs in relation to learning about critical thinking for health

We found that students are aware that critical thinking would help to make decisions about health for themselves and others (high confidence). Most students said that they search for health information on the Internet or ask their peers or family. Some said they could find out which treatments are better by trying them out and seeing what the effect was, or by asking friends or parents. Students shared their experiences of treatments they were familiar with for common conditions. Students commonly heard about treatments claims from peers, and that they generally accepted and believed them.

*“You can ask elders*, *your parents*, *your elder brothers/sisters*, *neighbours*, *and you know what they used which healed them quickly or you do research on Google*.*”*

**14-year-old student**

They had a general belief regarding what people can eat or drink to improve their health and which treatments they can use to improve common health conditions. Their beliefs about treatments were influenced by peers, the community, media and their families.

*“*… *when you are sick of flu or cough*, *you take ginger and lemon*, *you boil them*, *then you mix with honey*.*”*

**13-year-old student**

When we asked them whether it is important to learn critical thinking about health, they responded that it is important because it would give them confidence in their treatment choices. They also mentioned that knowing critical thinking, they can help themselves or others to make better choices. When we asked them how they can apply critical thinking about health in their daily lives, they said they would use medicines with caution and not accept every suggestion.

*“In order to avoid a person who can mislead you*, *because some can even give you wrong information on the treatment*, *Then when you take it without critical thinking*, *you have bad effect*, *which can even lead to death or you become disabled*.*”*

**14-year-old student**

### Current and expected ICT conditions

#### Policy and guidelines for use of ICT in teaching and learning

There are policy and guidelines in place that promote ICT use in teaching and learning (high confidence). The Government of Rwanda recognises ICT as a key pillar for national transformation. In 2016, the government approved the *ICT for education policy* [44]. The policy aimed to mobilise use of ICT in teaching and learning processes by developing ICT literacy and providing devices, connectivity, and digital content. In the education sector, ICT is regarded as a key strategy to drive teaching and learning.

REB has produced guidelines for establishing “smart classrooms” in schools to facilitate teaching and learning. Smart classrooms are computer laboratories with laptops, an Internet connection, and learning materials that develop 21^st^ century skills. There was an ICT implementation plan to provide all schools with smart classrooms by 2019.

*“Development and acquisition of digital content*, *aligned with the curriculum and that [*…*] is fully integrated with the use of ICT*, *[*…*] eventual shift from print to digital content as infrastructure is deployed in schools [*…*] Digital content has advantages of reducing costs of printing*, *distribution*, *replacement due to wear and tear and enriching the learning experience*.*”*

***ICT in education policy*, *page 4***.

#### Devices and connectivity for teaching and learning

The government of Rwanda has provided computers, connectivity and other ICT devices to more than 50% of schools for supporting teaching and learning (high confidence). According to the REB ICT for education department, over 50% of secondary schools in Rwanda have at least two smart classrooms and laptops for teachers in each department. Most schools have at least 100 computers for students and five computers for teachers in each department. The laptops are supplied by the government and have similar features, and the government pays for Internet access at the schools. Some schools have additional computers not supplied by the government. At the five schools we visited, there was also at least one data projector in the smart classrooms. Based on interviews with teachers, few students or teachers own a computer. Only one of the five schools we visited had some students who owned laptops.

#### Digital content for teaching and learning

There is an e-learning platform for schools that hosts non-interactive digital content in pdf formats. Some work is going on regarding interactive digital content (high confidence). All books developed for the competence-based curriculum are freely available. Interactive digital content is under development in pilot projects, according to the REB.

*“Well*, *we have not done so much on digital materials*, *what we have now is soft books in PDF*, *Digital content is different from soft content of the book because in digital content we should have animation*, *audio*, *Yeah*, *digital materials look like that*, *But we have that project*, *where we will make digital content for primary and secondary*.*”*

***Policymaker 3***

*“So far we have developed few interactive digital resources for each unit in a chapter*, *but we are now developing virtual labs*.*”*

***Policymaker 2***

#### Use of ICT for teaching and learning

Use of ICT for teaching and learning in Rwandan schools is limited due to limited ICT resources. Therefore, use of ICT in teaching is done in combination with traditional teaching (without ICT). Schools’ ICT facilities are available for teaching and learning on a rotating schedule, since there are not enough computers for all students to use at the same time (high confidence). In each school there is a timetable indicating when each class is scheduled to use a smart classroom. During breaks and weekends, smart classrooms at boarding schools are open for students to use. Students reported that their use of computers for teaching and learning outside of ICT classes occurs once or twice a week. Students use computers primarily for searching the Internet and for learning ICT skills. Teachers we interviewed reported that teaching and learning across subjects occurs mostly in classes without computers.

*“It might not always be possible for all classes to access smart classrooms in a bigger school but the need for it is weighed and classes are allowed accordingly*, *For boarding schools*, *they can even extend the learning hours to weekend program where students can have access to computers depending on the school timetable*.*”*

***Policymaker 3***

*Confidence in the findings*. Details of our assessment of confidence in the findings are summarised in the (S1 File). We judged that it is possible to have high confidence in all but one of the findings (which we rated as ‘moderate’).

## Discussion

The study aimed to explore the demand of teaching critical thinking about health conditions in Rwandan lower secondary schools using digital technology. We found that critical thinking is a key competence in Rwandan curriculum and health topics cut across different subjects. Furthermore students, teachers, and policy makers agreed there is a need for students to learn to think critically about health, and a need for learning resources to help teach critical thinking about health. We found that ICT devices and connectivity has already been supplied by the Rwanda Education Board to more than half of the schools in the country. However, use of ICT in daily teaching activities is limited by high computer to student ratios.

Internationally, there has been a shift towards competence-based curricula, and critical thinking is identified as a key competence in most curricula [16]. Critical thinking is a priority competence across subjects taught in lower secondary schools in Rwanda. However, critical thinking about health is not addressed explicitly and is not being taught. In the curricula, health is not a stand-alone subject, but health is included in three subjects: biology and health sciences, home science, and English. For English, health topics are used as a context for teaching English.

Teachers and curriculum developers did not express a direct ‘demand’ for these learning resources, likely because critical thinking about health is not explicitly described as a subject in the curriculum. However, both teachers and curriculum developers expressed a need for resources to help teachers teach critical thinking. We also uncovered opportunities in several subjects where teaching this content would fit with the existing curriculum.

Though critical thinking about health is not being taught, students recognise the importance of learning to think critically about health. They encounter many claims in their daily lives about the effects of health interventions and lack skills to critically appraise those claims. People have access to a massive amount of health information and need skills to know what is trustworthy [19].

We found that challenges to teaching critical thinking generally and critical thinking about health specifically include teachers’ lack of experience, training, and resources to help them. Similarly, a context analysis in Norway found that both critical thinking and health are emphasised in the curriculum, but teachers lack experience teaching critical thinking about health [45]. Other research has identified a lack of experience and training as a challenge to teaching critical thinking generally [46]. Our analysis suggests that to address these challenges, critical thinking learning resources should include support or training for teachers. In addition, because critical thinking and health are taught across subjects, resources are needed that can be used across subjects. If teaching critical thinking about health is distributed across subjects, teachers are likely to need a tool for coordinating this.

We also found challenges to using ICT for teaching and learning. Although more than half of the public schools in Rwanda now have smart classrooms, most schools have only two smart classrooms. This makes it hard to use them in daily teaching activities. Also, digital learning resources are limited to PDF textbooks provided by REB and available on their website. The use of digital learning resources, and particularly resources not provided by REB, is uncommon. Our results are similar to those of other studies which have found that barriers to using ICT for teaching and learning include poor infrastructure, lack of Internet connection, and sporadic electricity; teachers’ lack of competence, confidence, technological literacy, and pedagogical skills; and teachers’ perceptions and beliefs [47, 48]. Our findings suggest that close collaboration with policymakers—in Rwanda, the REB—is important in addressing these challenges, to ensure that digital learning resources are suitable for and integrated into the national platform, which would facilitate scaling up and sustaining use.

UNESCO has highlighted four mistakes to avoid when people want to integrate ICT in teaching and learning: “*installing learning technology without reviewing students’ needs and content availability*, *imposing technological systems from the top down without involving faculty and students*, *using inappropriate content from other regions of the world without customizing it appropriately*, *and producing low quality content that has poor instructional design and is not adapted to the technology in use*” [49]. This context analysis will help us to avoid those mistakes. In addition, we will develop learning resources iteratively, with continual in-depth feedback from students, teachers, and the curriculum committee.

### Strengths and limitations

A strength of this study is the use of multiple sources of data, including documents, interviews, focus group discussions, and observation. This provided a basis for triangulating the findings. In addition, data from our document review informed our collection of data from key informants’ interviews and focus group discussion. Another strength was the use of a modified version of CERQual to assess confidence in our findings.

A potential limitation is the possibility of social desirability bias among interview participants, particularly curriculum developers and teachers who teach critical thinking. They may have wanted to defend the extent to which critical thinking about health is covered in the curriculum and taught in Rwandan schools. We tried to mitigate this by emphasizing to all participants that we were not assessing the curriculum or teaching performance, but rather seeking to inform the development of our learning resources.

## Conclusion

This qualitative context analysis identified a need for learning resources to teach critical thinking about health to students in Rwanda. Students saw critical thinking about health as important for making better choices and are therefore likely to be motivated to engage in this learning. They are confronted with many claims about the effects of health interventions and recognize their need to know how to assess the trustworthiness of those claims. Critical thinking is a priority competence in the Rwandan curriculum. However, teachers need support for teaching critical thinking skills generally, and critical thinking about health specifically. Experience from elsewhere suggests that digital learning resources can reduce costs compared to printed material, and interactive resources may have additional advantages. However, widespread use and sustainability of digital learning resources depends on support from the Rwanda Education Board. Resources also need to be designed in a way that makes them adaptable for use in schools with limited ICT resources, as well as suitable for use by teachers with limited ICT experience.

## Supporting information

S1 File CERQUAL assessment of key findings for context analysis. (DOCX)
