# Supplementary material for: Teaching critical thinking about health using digital technology in lower secondary schools in Rwanda: A qualitative context analysis
Source: PLoS One. 2021 Mar 22;16(3):e0248773. doi: 10.1371/journal.pone.0248773 (PMC7984628; doi:10.1371/journal.pone.0248773)
Supplement: S1 File — (DOCX) [file pone.0248773.s001.docx]

| **Findings** | **CERQUAL ASSESSMENT** | | | |  |
| --- | --- | --- | --- | --- | --- |
| **Demand for learning resources to teach critical thinking about health** | ***Relevance (the contexts of the data sources underlying a study finding are substantively different from the context of the study question)*** | ***Methodological limitations (the data sources underlying a finding are shown to have problems in the way the data were collected)*** | ***Coherence (Does the finding provide a convincing explanation for the patterns found in the data-the fit between the data from your sources and the study finding is not completely clear)*** | ***Adequacy (the data underlying a study finding are not sufficiently rich or only come from a small number of participants)*** | ***Overall CERRQUAL assessment*** |
| The competence-based curriculum demands developing generic competences including critical thinking, research and problem solving in all subjects | There was no concerns regarding relevance of data sources (document review, school teachers and curriculum specialists participants) and the finding. | There was no methodological limitations related to data sources, data collecction process or researcher reflexivity | There was coherence of the three sources of data to the finding | There was sufficient data supporting the finding | High |
| Health related topics taught in secondary school subjects provide opportunity for developing competences for critical thinking about health among learners | There was no concern related to the relevance of the finding across participants and sources of data | There was no issues of methodological limitations | There was a general coherence of the finding within the three sources of data (document review, interviews and focus group discussion | There was sufficient data supporting the finding | High |
| The current curriculum lays out the demand for development of new textbooks and teachers’ guides to facilitate a learner-centred approach | There was no concerns regarding relevance of findings across participants and sources of data | There was no issues of methodological limitations | There was a general coherence of the finding within the three sources of data (document review, interviews and focus group discussion | There was sufficient data supporting the finding | High |
| Understanding and developing critical thinking about health varies among teachers | There were concerns related to teachers interviewed. We did not include teachers from rural schools due to COVID-19 outbreak | There was no issues of methodological limitations | There was coherence of the finding across study participants | There was sufficient data supporting the finding | Moderate |
| We found that students are aware that critical thinking would help to make decisions about health for themselves and others | There is was no concerns related to relevance of the finding to the participants data | There was no methodological concerns for the finding | There was coherence of the finding across study participants | There was sufficient data supporting the finding | High |
| At the end of lower secondary school, students should be able to apply science in advocating for personal, family and community health. | The were no concerns related to the relevance of the finding to the data sources-document review | There was no methodological concerns for the finding | There was coherence of data across documents reviewed | There was sufficient data supporting the finding | High |
| **Current and expected ICT conditions for teaching/learning purposes in secondary schools** |  |  |  |  |  |
| There are policy and guidelines in place that promote ICT use in teaching and learning | There are policy and guidelines in place that promote ICT use in teaching and learning | There was no methodological limitations related to data collection process | The finding has coherence of data across documents reviewed | There was sufficient data supporting the finding | High |
| The government of Rwanda provided to more than 50% of schools with computers, connectivity and other ICT devices to support teaching and learning | The government of Rwanda provided to more than 50% of schools with computers, connectivity and other ICT devices to support teaching and learning | There was no methodological limitations related to data collection process | The finding has coherence of data across multiple sources | There was sufficient data supporting the finding | High |
| Public schools have smart classrooms with at least two rooms containing 50 computers each, provided by the government. Very few teachers and students own their computers. | Public schools have smart classrooms with at least two rooms containing 50 computers each, provided by the government. Very few teachers and students own their computers. | There was no methodological limitations related to data collection process | The finding has coherence of data across multiple sources | There was sufficient data supporting the finding | High |
| There is an e-learning platform for schools that hosts non-interactive digital content in pdf formats. Some work is going on regarding interactive digital content | There is an e-learning platform for schools that hosts non-interactive digital content in pdf formats. Some work is going on regarding interactive digital content | There was no methodological limitations related to data collection process | The finding has coherence of data across multiple sources | There was sufficient data supporting the finding | High |
| Schools’ ICT facilities are available for teaching and learning on a rotating schedule, since there are not enough computers for all students to use at the same time. | Schools’ ICT facilities are available for teaching and learning on a rotating schedule, since there are not enough computers for all students to use at the same time. | There was no methodological limitations related to data collection process | The finding has coherence of data across multiple sources | There was sufficient data supporting the finding | High |
